# High-Temperature Cyclic Oxidation Behavior and Microstructure Evolution of W- and Ce-Containing 18Cr-Mo Type Ferritic Stainless Steel

**DOI:** 10.3390/ma17102230

**Published:** 2024-05-09

**Authors:** Jiahao Zheng, Yang Feng, Yang Zhao, Liqing Chen

**Affiliations:** 1 State Key Laboratory of Rolling and Automation, Northeastern University, Shenyang 110819, China; aminee@163.com (J.Z.); 13199587276@163.com (Y.F.); 2 School of Materials Science and Engineering, Northeastern University, Shenyang 110819, China; zhaoyang0323@smm.neu.edu.cn

**Keywords:** ferritic stainless steel, cerium, tungsten, cyclic oxidation behavior, oxidation mechanism

## Abstract

Due to the recurrent starting and stopping operations of automobiles during service, their engines’ hot ends are continually subjected to high-temperature cyclic oxidation. Therefore, it is crucial to develop ferritic stainless steels with better high-temperature oxidation resistance. This study focuses on improving the high-temperature cyclic oxidation performance of 18Cr-Mo (444-type) ferritic stainless steel by alloying with high-melting-point metal W and the rare earth element Ce. For this purpose, a high-temperature cyclic oxidation experiment was designed to simulate the actual service environment and investigate the high-temperature cyclic oxidation behavior and microstructure evolution of 444-type ferritic stainless steel alloyed with W and Ce. The oxide structure and composition formed during this process were analyzed and characterized using scanning electron microscopy/energy dispersive spectroscopy (SEM-EDS) and electron probe X-ray micro-analyzer (EPMA), in order to reveal the mechanism of action of W and Ce in the cyclic oxidation process. The results show that 18Cr-Mo ferritic stainless steel alloyed with W and Ce exhibits an excellent resistance to high-temperature cyclic oxidation. The element W can promote the precipitation of the Laves phase between the matrix and the oxide film, and the small-sized Laves phase can inhibit the interfacial diffusion of oxidation reaction elements and prevent the inward growth of the oxide film. The element Ce can refine oxide particles and reduce the thickness of the oxide film. CeO_2_ particles within the oxide film can serve as nucleation sites for the formation of oxide particles from reactive elements, and they also contribute to pinning the oxide film, thereby enhancing its adhesion.

## 1. Introduction

The thermal conductivity of ferritic stainless steel is 130–150% of that of austenitic stainless steel, and its thermal expansion coefficient is 60–70% of that of austenitic stainless steel [1]. This makes it suitable for applications in heat exchange and thermal cycling environments, such as automotive exhaust pipes and solid oxide fuel cell (SOFC) frameworks [2]. In the hot end of automotive exhaust systems, 444 ferritic stainless steel is widely used due to its good high temperature oxidation resistance and low thermal expansion coefficient. With the continuous upgrading of energy conservation and emission reduction standards in the automotive industry, the hot end of the automotive exhaust system needs to withstand higher temperatures. Therefore, there is an urgent need for a new type of heat-resistant ferritic stainless steel with excellent high-temperature oxidation and corrosion resistance and economic efficiency.

In order to obtain heat-resistant ferritic stainless steel with excellent oxidation resistance and considering the above requirements, some research studies have been conducted on related ferritic stainless steel through alloying. These studies have shown that the addition of the alloying element W enhances the high-temperature fatigue performance through precipitation or solid solution strengthening [3] and significantly increases the high-temperature tensile strength [4]. Furthermore, adding alloying elements such as W can improve the thermal mechanical fatigue performance of medium chromium stainless steel [5]. In the study by Yun et al. [6], it was found that the element W can affect the high-temperature oxidation resistance of Fe-22Cr-0.5Mn ferritic stainless steel in the temperature range of 700 to 900 °C. Safikhani et al. [7] found that adding elements Ti and W to Fe-22Cr-0.5Mn ferritic stainless steel reduced the oxidation rate of ferritic stainless steel at 900 °C. The study by Wei et al. [8] showed that by adding element W to 18Cr ferritic stainless steel, the Laves phase in the oxide film can effectively hinder the internal oxidation process and improve its high-temperature oxidation resistance in the water vapor environment at 950 °C.

Adding rare earth elements to ferritic stainless steel is usually considered an effective way to improve its high-temperature oxidation resistance and hot corrosion resistance. Specifically, the rare earth element Ce can refine oxide particles during isothermal oxidation [9], and it can have a positive impact on the high-temperature oxidation resistance of ferritic stainless steel by enhancing the adhesion or inhibiting the exfoliation of the oxide film [10]. The work of Ramanathan et al. [11] shows that by adding rare earth elements to Fe-Cr and Ni-Cr alloys, the growth mechanism of their oxide films is altered to reduce the alloy oxidation rate. The existing research on using rare earth elements to improve the high-temperature oxidation resistance of steel is concentrated below 1000 °C. Numerous studies have shown similar results; that is, within this temperature range, the high-temperature oxidation resistance of steel with the addition of rare earth elements can be improved. Li et al. [12] found that an increase in the content of rare earth elements can improve the rupture strength of the oxide layer through the study of 00Cr17NbTi ferritic stainless steel containing Ce. Thanneeru et al. [13] coated stainless steel surfaces with cerium nanocrystalline coatings and found that oxygen vacancies in the cerium dioxide nanocoatings are beneficial to the formation of the chromium protective layer. In terms of mechanism research, many scholars have proposed different hypotheses. Silva et al. [14] reduced the interfacial segregation of reaction elements by adding the Ce element to Fe-Mn-Si-Cr-Ni stainless steel, inhibited the generation and fragmentation of oxide nodules, and improved its antioxidant performance. The study by Alman et al. [15] suggests that Ce in Fe-22Cr-0.5Mn primarily protects the surface of ferritic stainless steel by forming a continuous Ce oxide layer on the surface. You et al. [16] used the Ce element to alter the growth mechanism of the Cr_2_O_3_ oxide layer on the surface of 430 ferritic stainless steel, transforming it from an outward diffusion of Cr^3+^ ions to an inward diffusion of O^2−^ ions, thereby reducing the oxidation rate of stainless steel.

Upon reviewing the available research, it becomes evident that various conjectures about the role of rare earth elements predominantly concentrate on specific steel types, with limited exploration at temperatures exceeding 1000 °C. There is almost no research on the cyclic oxidation of stainless steel among them. Nevertheless, in practical service scenarios, the cyclic oxidation performance of stainless steel imposes stricter requirements than those for isothermal oxidation. To exhibit a superior resistance to high-temperature cyclic oxidation, ferritic stainless steel must possess a low oxidation rate and ensure strong adhesion between the oxide layer and the substrate. To enhance the high-temperature performance of ferritic stainless steels, a novel modified steel has been designed. Studies have shown that by regulating the high-temperature precipitation behavior of the Laves phase through the judicious addition of the W and Ce elements, both the high-temperature strength [17] and hot-working properties [18] of the ferritic stainless steel can be significantly improved. What is more, the addition of Ce and W increases the pitting potential of ferritic stainless steel and enhances its corrosion resistance [19]. In this study, the modified heat-resistant ferritic stainless steel containing W and Ce was used as the experimental material to study the effect of W and Ce on the high-temperature cyclic oxidation behavior of ferritic stainless steel. Under this condition, the underlying mechanism of high-temperature cyclic oxidation resistance in ferritic stainless steel is elucidated by studying the composition and structure of the oxide film. This study aims to provide a theoretical foundation and guidance for the compositional design, optimization, and service performance of this heat-resistant ferritic stainless steel.

## 2. Experimental

In this study, four experimental steels with different chemical compositions were designed to improve the high-temperature oxidation resistance of 444-type ferritic stainless steel by adding appropriate amounts of the W and Ce elements. The chemical compositions of the experimental steels were analyzed by ICP-OES (Optima 8000DV PerkinElmer, Waltham, MA, USA) and listed in Table 1. Among them, 444-Ce steel is obtained by adding 0.05% of the element Ce to 444-type ferritic stainless steel; 444-0.5W-Ce and 444-1W-Ce were obtained by adding 0.5% and 1% of the element W on the basis of F2 steel. These experimental steels were first melted and cast into steel ingots in a 150 kg capacity vacuum induction furnace (Jinzhou Electric Furnace Co., Ltd., Jinzhou, China), followed by forging the ingots into 42 mm × 180 mm × 800 mm billets. After being held at 1200 °C for 3 h, the forged billets were hot rolled into 4 mm-thick slabs in 8 passes, and finally cold rolled into 1 mm-thick slabs on a reversible two-roll rolling mill. The cold-rolled sheet was annealed at 1050 °C for 5 min and cooled to room temperature. The samples for the cyclic oxidation experiment with a size of 25 mm × 20 mm × 1 mm were cut out of the cold rolled sheet. In order to simulate the actual service conditions of ferrite stainless steel in the hot end of the automotive exhaust system under alternating heating and cooling cycles, a cyclic oxidation experiment was designed in this study. The experimental steel samples with different compositions were oxidized in a resistance furnace at 950 °C, 1050 °C, and 1100 °C for 1 h, followed by air-cooling to room temperature and completing one cycle. In this way, multiple cycles were performed. The high-temperature oxidation behavior of these steels was thoroughly evaluated, and their oxide layer microstructures were meticulously characterized.

All samples for cyclic oxidation experiments were polished with 1200# sandpaper, then washed in anhydrous ethanol by using an ultrasonic cleaner before being dried. The cleaned samples were placed in a crucible and weighed. Each test was performed after 20, 50, 80, 120, and 200 cycles of oxidation for each group of samples. The weight gain in oxidized samples per unit area and the spalled mass of the oxidized film per unit area were measured and calculated by means of an electronic balance with accuracy of 0.1 mg (ME204E, METTLER, Shanghai, China), and the kinetic curve of cyclic oxidation was plotted based on these measurements. The microstructure, cross-sectional morphology of the oxide film, and elemental distribution of the samples after cyclic oxidation were characterized using a field emission electron probe (JXA-8530F, JEOL, Tokyo, Japan), and the precipitates were quantitatively analyzed using an electron spectrometer (INCA-EDS, Oxford Instruments, Oxford, UK). The entire morphology of the surface oxide film and the morphology and density of local oxide particles in each sample were observed and analyzed by scanning electron microscope (ULTRA 55, Zeiss, Oberkohen, Germany). The phases formed on the oxidized surfaces were analyzed by an X-ray diffraction (MPDDY2094, PANalytical B.V., Almelo, The Netherlands), with a diffraction angle set at 20~90°, a scanning step set at 0.02°, a voltage of 40 kV, and a current of 45 mA. The XRD results were analyzed using Jade 6 software (6.05.0026) to distinguish the type of oxide in the oxide film.

## 3. Results and Discussion

### 3.1. Initial Microstructure

**Figure 1:**
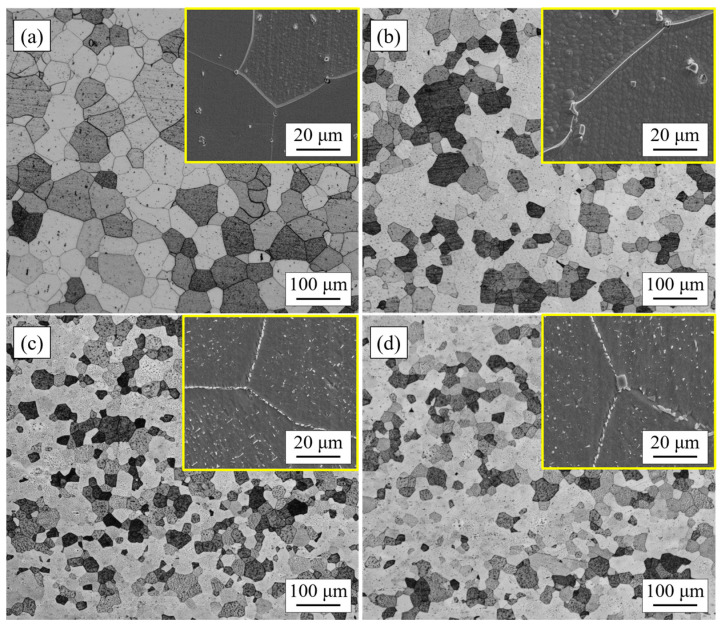
Optical micrographs of experimental steels annealed at 1050 °C for 5 min after cold rolling. (**a**) 444; (**b**) 444-Ce; (**c**) 444-0.5W-Ce; (**d**) 444-1W-Ce.

Figure 1 shows the optical micrographs of four types of ferrite stainless steels after cold rolling and annealing at 1050 °C for 5 min, which was the initial structure of the four types of ferrite stainless steels before the cyclic oxidation experiment. It can be observed that all four stainless steels were composed of polygonal ferrite and a certain number of precipitates. According to the cross-sectional method, the average grain sizes of 444, 444-Ce, 444-0.5W-Ce, and 444-1W-Ce steels were 93 μm, 71 μm, 63 μm, and 61 μm, respectively. By scanning electron microscopy observation, it can be observed that a large number of precipitates formed at the grain and grain boundaries of stainless steel after the addition of W. These precipitates can pin the grain boundaries and reduce the grain coarsening rate. In addition, the precipitate size in 444-1W-Ce steel was slightly larger than that in 444-0.5W-Ce steel.

### 3.2. Oxidation Kinetics

**Figure 2:**
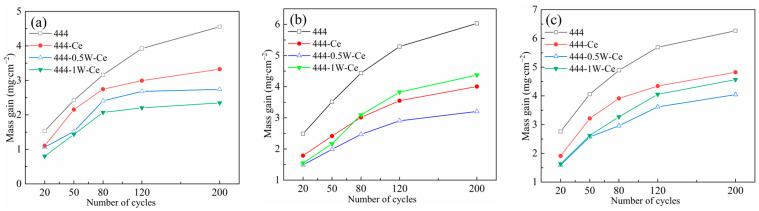
Oxidation weight gain per unit area of four stainless steels after 200 cycles of cyclic oxidation in air at different temperatures: (**a**) 950 °C; (**b**) 1050 °C; (**c**) 1100 °C.

Figure 2 shows the weight gain curves per unit area for the four experimental steels after 200 cycles of oxidation at 950 °C, 1050 °C, and 1100 °C. As the number of cycles of oxidation increases, the weight of the oxide layer continues to increase. The shape of its weight gain curve is similar and conforms to the parabolic oxidation law, indicating that the high-temperature cyclic oxidation of the experimental steel in air belongs to the diffusion controllable type [20], and has a good high-temperature oxidation resistance. The difference in the weight gain per unit area of the experimental steels was not obvious when the cyclic oxidation temperature was 950 °C. After adding the element Ce, the oxidation weight gain of 444-type ferritic stainless steel decreased from 4.55 mg/cm^2^ to 3.32 mg/cm^2^. After adding 0.5% or 1% W, the oxidation weight gain of ferritic stainless steel further decreased to 2.73 mg/cm^2^ and 2.34 mg/cm^2^, respectively. These results indicate that Ce and W can effectively improve the high-temperature cyclic oxidation resistance of ferritic stainless steel. With increasing the oxidation temperature, the oxidation reaction rate of these experimental steels increases as well. When the temperature of cyclic oxidation reaches 1050 °C and 1100 °C, the weight gain of these four types of ferrite stainless steel during the initial stage of oxidation reaction (within 50 cycles of oxidation) was consistent with that at 950 °C. As the number of cyclic oxidation reactions further increases, the oxidation weight gain of 444-1W-Ce steel suddenly increases and exceeds that of 444-0.5W-Ce steel. This indicates that when the content of W element is excessive, the effect of W element on improving the high-temperature oxidation performance of ferritic stainless-steel decreases, which is not conducive to the stability of its high-temperature oxidation resistance during long-term service at high temperatures. Due to the fact that the element W does not directly participate in the oxidation reaction, its effect on high-temperature oxidation resistance mainly comes from the precipitation of the element W at the interface of the matrix/oxide film. Based on Figure 2, it can be inferred that this is likely due to the precipitation and coarsening of the Laves phase precipitated by the W element at the interface between the matrix and oxide layer.

**Figure 3:**
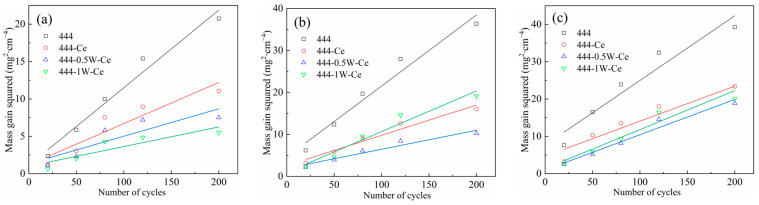
Oxidation kinetics of four stainless steels after 200 cycles of cyclic oxidation in air at different temperatures: (**a**) 950 °C; (**b**) 1050 °C; (**c**) 1100 °C.

In order to further analyze the high-temperature cyclic oxidation kinetics of these four types of stainless steel, the oxidation reaction rate constants of these steels should be fitted and calculated based on their oxidation weight gain data. Figure 3 shows the oxidation kinetics for the four experimental steels after 200 cycles of oxidation at 950 °C, 1050 °C, and 1100 °C. The parabolic law for the oxidation reaction can be expressed by Formula (1) [21]:

(1) $${\left(\Delta m/s\right)}^{2}=C+k_{p}t$$

where ∆*m/s* refers to the weight gain per unit area in mg/cm^2^, *t* represents the oxidation time with unit s, *k_p_* corresponds to the oxidation reaction rate constant in mg^2^∙cm^−4^∙s^−1^, and C is a constant.

Table 2 lists the oxidation rate constants of these four types of stainless steel after cyclic oxidation in air at different temperatures. It can be seen that the trend of oxidation weight gain of these four types of stainless steel conforms to the parabolic law of the oxidation weight gain curve. When high-temperature cyclic oxidation is carried out, the high-temperature cyclic oxidation rates of 444-type ferritic stainless steels decrease with the addition of Ce and W. When the cyclic oxidation temperature is 1050 °C, the oxidation rate of 444 ferrite stainless steel is 1.87 × 10^−2^ mg^2^∙cm^−4^∙s^−1^, while the oxidation rates of 444-Ce and 444-0.5W-Ce ferritic stainless steels are only 3.07 × 10^−3^ mg^2^∙cm^−4^∙h^−1^ and 2.76 × 10^−3^ mg^2^∙cm^−4^∙h^−1^, respectively. The elements W and Ce exhibit inhibitory effects on the oxidation process of 444 ferritic stainless steel and the inhibition effects depend on the combined influence of the elements Ce and W on the diffusion rate of reactive elements on the surface of ferritic stainless steels, the oxide particle microstructure, and the adhesion of the oxide film. Table 2 also lists the oxidation rate constants for several other stainless steels when oxidized at high temperatures. In the range of 900–1000 °C, these steels have a similar high-temperature oxidation resistance to 444-type ferritic stainless steels, especially Crofer 22H steel, which also contains W and Nb. Compared with 444 ferritic stainless steel, 441 ferritic stainless steel does not contain Mo, and its oxidation rate coefficient is as high as 2.16 × 10^−3^ at 950 °C.

The relationship between the oxidation rate and oxidation activation energy of ferritic stainless steel can be expressed using the Arrhenius Formula (2) [18]:

(2) $$kp=Aexp\left(-Q/RT\right)$$

where *k_p_* refers to the oxidation reaction rate constant in mg^2^∙cm^−4^∙s^−1^, *T* represents the oxidation temperature with unit °C, *Q* corresponds to the oxidation activation energy of experimental steel in kJ∙mol^−1^, R represents the universal gas constant, and A is a constant.

Table 3 lists the oxidation activation energy of experimental steel in different temperature ranges. The oxidation activation energy of ferritic stainless steel containing the W and Ce elements is higher than that of 444 type ferritic stainless steel. This indicates that in the initial stage of oxidation, 444 type ferritic stainless steel is more prone to oxidation. The oxidation activation energies of 444 and 444-0.5W-Ce ferritic stainless steel in the range of 950 °C to 1100 °C are 246 kJ∙mol ^−1^ and 292 kJ∙mol^−1^, respectively.

### 3.3. Surface Morphology of the Oxide Film

**Figure 4:**
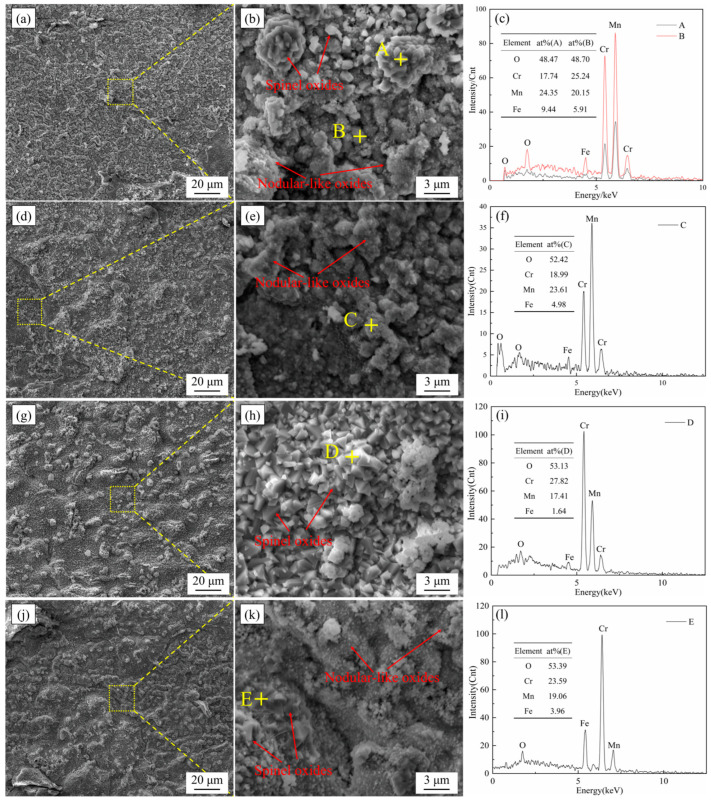
The surface morphology and EDS of the oxide film of four types of ferritic stainless steels after cyclic oxidation in air at 1050 °C for 200 cycles. (**a**–**c**) 444; (**d**–**f**) 444-Ce; (**g**–**i**) 444-0.5W-Ce; (**j**–**l**) 444-1W-Ce.

The structural characteristics of the oxide film generated by ferritic stainless steel in the high-temperature corrosion environment can reflect the high-temperature oxidation resistance of ferritic stainless steel to a certain extent. Figure 4 shows the surface morphology of the oxide film for the four types of ferritic stainless steels after cyclic oxidation in air at 1050 °C for 200 cycles. It can be found that there were two main types of surface oxides of ferritic stainless steel when we use scanning electron microscope to observe the morphology of the oxide film. The oxide film on the surface of ferritic stainless steel was composed of nodular-like oxides formed by the aggregation of small oxide particles and another spinel oxides with regular shape. Both types of the surface oxides were Cr-Mn type oxides and the nodular-like oxides have a relatively higher content of Mn. Nodular-like oxides are mostly spherical or strip shaped. The surface of the oxide film formed by these oxides has many grooves and roughness, and there are many voids between the oxides. Spinel type oxides have a relatively dense structure, which can effectively inhibit the inward diffusion of oxygen elements and provide better protection for the surface of ferritic stainless steel. It can be observed that the surfaces of 444 and 444-Ce ferritic stainless steels have more nodular-like oxides, while the proportion of spinel oxides in the surface oxides of the W-containing ferritic stainless steels was increased. In addition, it can be found that the addition of the Ce element significantly reduces the volume of nodular oxide on the surface of 444-type ferritic stainless steel.

Due to the difference in thermal expansion coefficients between the metal material matrix and the oxide film, the oxide film is subjected to thermal and growth stresses during cyclic use in alternating cold and hot environments. If the stress cannot be effectively released, the oxide film will crack and spall off after the stress reaches a certain limit. After the oxide film is spalled off, the location of the spalling will expose the metal matrix and the metal substrate without the protection of the oxide film will quickly oxidize in a high-temperature environment. Compared to conventional isothermal oxidation, an excellent resistance to high-temperature cyclic oxidation not only requires the alloy to have the ability to resist high-temperature oxidation, but also its oxide layer needs to have a good resistance to spalling. When the oxide film cracks to release stress, the reaction elements will diffuse inward through the crack and form new oxides inside the oxide film. These oxides may either lift the outer oxide film and cause it to spall off or fill the cavities of the cracks to repair the oxide film. Sabioni et al. [26] showed that when cooled in air, the oxide film releases stress through creep and other means and the oxide particles with regular shapes or smooth surfaces are more likely to release thermal and growth stresses inside the oxide layer by sliding against each other. Compared to nodular oxides, spinel type oxides have a smoother surface and are more conducive to the release of stress in the oxide film.

**Figure 5:**
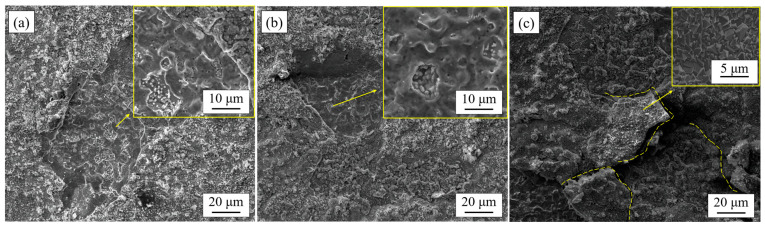
Micro-morphology of the spalling areas on the surface of the oxide film of several 444-type ferritic stainless steels after cyclic oxidation in air at 1050 °C for 200 cycles. (**a**) 444; (**b**) 444-Ce; (**c**) 444-1W-Ce.

Figure 5 shows the morphologies of the surface spalling areas of the oxide layer of 444, 444-Ce, and 444-1W-Ce ferritic stainless steels after 200 cycles of oxidation in air at 1050 °C. The difference in affinity of alloy elements for oxygen ions determines the sequence of binding in the initial stage of oxidation reaction. The affinity of different alloy elements for oxygen ions is Si > Mn > Cr > Fe [27]. After the oxide film spalls off, the metal matrix will continue to oxidize inward and form pits, as shown in Figure 5a. Observation of the oxide particles in the pits at a higher magnification shows that these particles tend to form nodular oxides, as shown in Figure 5b. It can be inferred that due to the double oxide film structure of 444-type ferritic stainless steel, the diffusion rate of the element Mn in the oxide film will decrease when the inner oxide film was formed. As a result, the surface will be more inclined to form spinel oxides during the following oxidation process. The adhesion between spinel oxides was stronger and the tendency of the oxide film to spall off was lower. The surface of spinel oxides was smooth and regular in shape, with small gaps between the oxides. When cracks or cavities occur, new oxide particles can be formed quickly to fill up the cavities to avoid the oxide film from spalling off after cracking. It can be seen from Figure 5c that most of the oxide films are mainly composed of spinel oxides which can keep a certain adhesion after cracking without spalling off.

**Figure 6:**
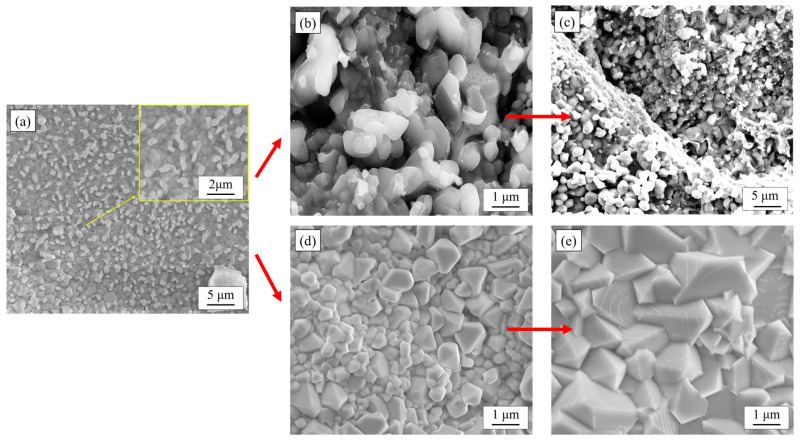
Micro-morphology of two kinds of oxide particles formed on the surface of ferritic stainless steel at the early stage of oxidation (0–5 min). (**a**) Fine oxides formed in the outer film of the oxide film; (**b**) New oxide particles nucleated on the surface of the oxide particles after they have grown to a certain extent; (**c**) Fine oxide particles were stacked into nodular-like oxides; (**d**) Fewer new oxide particles were formed on the surface of the oxide particles; (**e**) The oxide particles grew to form spinel oxides.

From the SEM micrographs, there were differences in the growth patterns of the nodular-like oxides and the spinel oxides. By taking a large number of photographs of the morphology of the ferritic stainless-steel surface at the early stage of oxidation (0–5 min), it was found that the structural differences between the nodular and spinel oxides were strongly related to the growth rate and shape of the oxide particles. As the growth of oxide particles was limited, smaller oxide particles stack with each other and eventually form ellipsoidal or strip-shaped nodular-like oxides, as shown in Figure 6c. Because the rare earth elements are more active than the oxide film forming elements, the rare earth oxide particles formed on the surface of Ce-containing 444-type stainless steel can provide nucleation sites for the main reaction elements of the oxide film. These rare earth oxides promote the formation of more oxide particles and play a role in refining the oxide particles. In addition, Ce-containing oxides can also have a pinning effect on the oxide film, improving its density and adhesion through bonding. The unrestricted growth of oxides will gradually form spinel type oxides, and these spinel surfaces will slowly continue to grow outward, but do not promote the nucleation of new oxides. Eventually, these spinel oxides gradually squeezed together to form a very dense oxide film. The Cr_2_O_3_ oxide film is unstable at high temperature and can easily produce volatile CrO_3_, leading to the decrease in Cr content in the oxide film. The Cr-Mn oxide film can effectively reduce the volatile loss of element Cr and maintain the stability of the inner oxide film [28].

**Figure 7:**
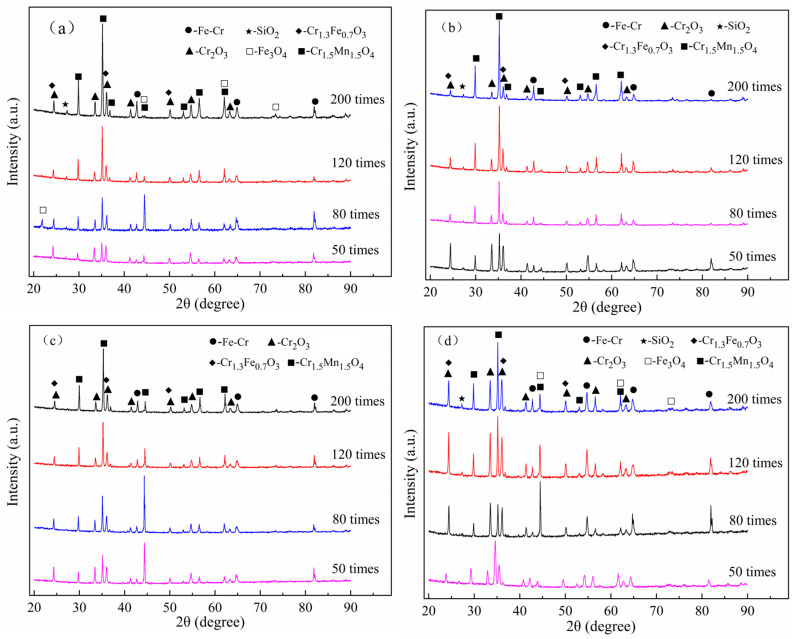
The oxidized products of the experimental steels after cyclic oxidation at 1050 °C for 50, 80, 120, and 200 cycles: (**a**) 444; (**b**) 444-Ce; (**c**) 444-0.5W-Ce; (**d**) 444-1W-Ce.

The oxidation products of the experimental steels after cyclic oxidation at 1050 °C for 50, 80, 120, and 200 cycles were determined and analyzed by XRD, and the results are shown in Figure 7. The main products of the four 444-types ferritic stainless steels after cyclic oxidation in air at 1050 °C were Cr_2_O_3_ (ICDD-PDF NO. 38-1479) and Mn_1.5_Cr_1.5_O_4_ (ICDD-PDF NO. 75-1614). In addition, the diffraction peaks of Cr_1.3_Fe_0.7_O_3_ (ICDD-PDF NO. 35-1112), Fe_3_O_4_ (ICDD-PDF NO. 86-1351), and SiO_2_ (ICDD-PDF NO. 86-1630) also appeared in the specimens of these stainless steels after oxidation. Due to the spalling phenomenon of the oxide layer during temperature cycling, the presence of Fe-Cr diffraction peaks in the matrix can be found by detecting these spalling parts. It can be clearly observed from Figure 7d that the 444-1W-Ce steel exhibits stronger Fe-Cr diffraction peaks at 80 and 120 cycles. This indicates that the rapid increase in the oxidized weight gain rate of 444-1W-Ce ferrite stainless steel in air at 1050 °C exceeding that of 444-Ce ferrite stainless steel is most likely due to more defects in the oxide layer of 444-1W-Ce at high temperatures.

### 3.4. Cross-Sectional Morphology of Oxide Film

**Figure 8:**
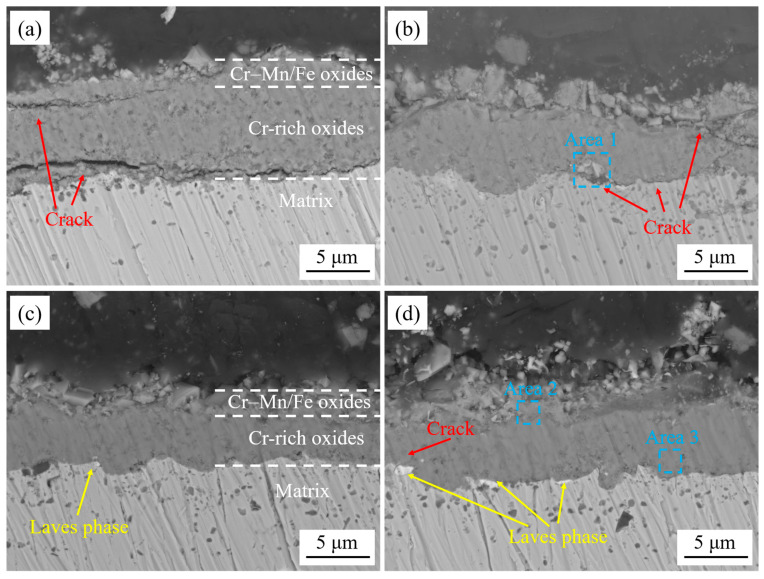
The cross-sectional morphology of the oxide film of the experimental steels after cyclic oxidation in air at 1050 °C for 50 cycles: (**a**) 444; (**b**) 444-Ce; (**c**) 444-0.5W-Ce; (**d**) 444-1W-Ce.

Figure 8 shows the cross-sectional morphology of the oxide film of the experimental steels after cyclic oxidation in air at 1050 °C for 50 cycles. It can be observed that the thickness of the oxide film of 444-type ferritic stainless-steel decreases slightly after the addition of W and Ce, and the adhesion between the matrix and the oxide film is improved. In 444-type ferritic stainless steel, there are very obvious cracks and cavities at the interface between the substrate and the oxide layer. These transverse cracks continue to extend, and once the internal thermal stress of the oxide layer is too large to form longitudinally through microcracks, the oxide layer will spall off. There were a small number of cavities inside the 444-Ce stainless steel which were surrounded by fine microcracks. According to the EDS results (Area 1 in Table 4), it can be found that there were Si oxides produced in these cavities. A recent study [29] has shown that Si oxide is an extremely stable oxide that tends to form at the interface and can inhibit cation transport at the interface between the matrix and oxide film, thereby reducing the oxidation rate of the alloy. However, these oxides can easily form discontinuous cavities and even exacerbate the spalling of the Cr_2_O_3_ oxide film in severe cases. The adhesion between the oxide film and the matrix was better for the 444-0.5W-Ce and 444-1W-Ce stainless steels. There is a slight increase in the quantity of Laves phases present at the interface between the matrix and the oxide film after the addition of W. The occurrence of numerous serrated matrix/oxide interfaces adjacent to the locations of Laves phase precipitation can be attributed to the inhibitory effect of these phases on the inward growth of the inner oxide layer, causing the oxide film to extend downward by bypassing the precipitates.

**Figure 9:**
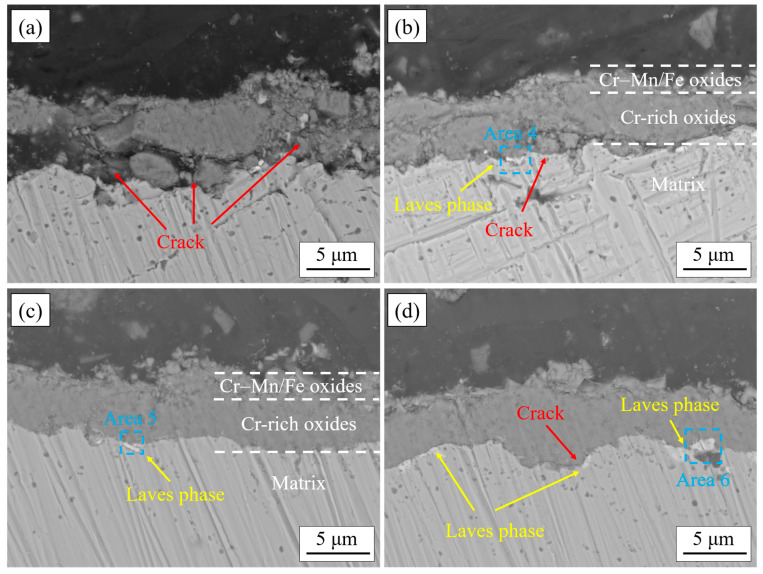
The cross-sectional morphology of the oxide film of the experimental steels after cyclic oxidation in air at 1050 °C for 200 cycles: (**a**) 444; (**b**) 444-Ce; (**c**) 444-0.5W-Ce; (**d**) 444-1W-Ce.

Figure 9 shows the cross-sectional morphology of the oxide film of the experimental steels after cyclic oxidation in air at 1050 °C for 200 cycles. Compared with the cross-section of the oxide layer after 50 cycles, after 200 cycles of high-temperature oxidation, there were more internal defects in the oxide layer of 444 and 444-Ce ferritic stainless steel, especially the oxide layer of 444 ferritic stainless steel which was severely broken. Inside the oxide film of 444-Ce ferritic stainless steel, there were some oxide films that were broken but not spalling. It can be observed that many new oxides formed in the cavities of the oxide film, which could fill the cavities and cracks and finally repair the defects of the oxide film [30]. The 444-0.5W-Ce ferritic stainless steel has a superior adhesion between the oxide film and the matrix. Except for a small number of cavities, there were no obvious defects in its oxide film. Large-sized Laves phases precipitated at the interface between the matrix and oxide film of 444-1W-Ce ferrite stainless steel, and these continuously precipitated Laves phases would increase the thermal cracking tendency of the oxide film and promote the separation of the oxide film from the matrix [31]. Table 4 shows that the Laves phase precipitated at the interface between the matrix and oxide film of the W-containing 444-type ferritic stainless steel was transformed from Fe_2_(Nb, Mo) to Fe_2_(Nb, Mo, W).

**Figure 10:**
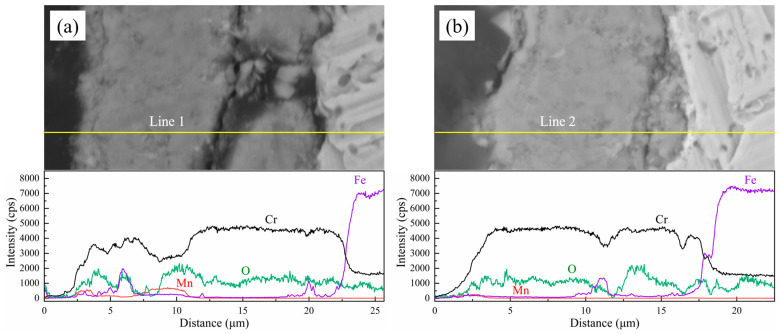

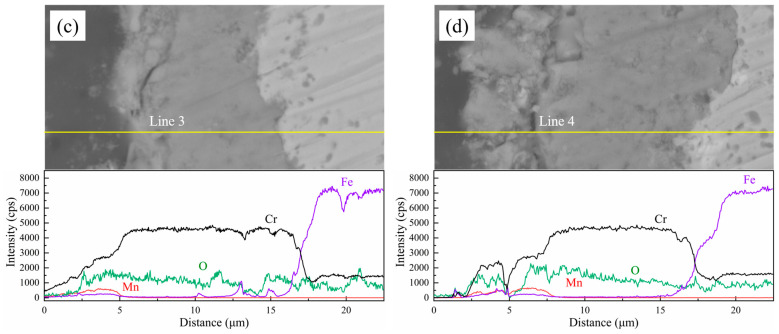
Sectional morphologies and the corresponding line scanning analysis of the oxide layers of the experimental steel after cyclic oxidation in air at 1050 °C for 200 cycles: (**a**) 444; (**b**) 444-Ce; (**c**) 444-0.5W-Ce; (**d**) 444-1W-Ce.

Figure 10 shows the sectional morphologies and its line scanning analysis of the oxide layers of the experimental steels after cyclic oxidation in air at 1050 °C for 200 cycles. As indicated in the results of the line scanning analysis, the oxide films of all four types of stainless steels were mainly composed of Cr-Mn oxides in the outer layer and Cr oxides in the inner layer. The thickness of the oxide film of 444-type ferritic stainless steel was greater than that of the other three ferritic stainless steels. The content of the Fe element in each group of specimens was low along the entire thickness of the oxide film, but increased sharply at the interface between the oxide film and the matrix. In other words, the Cr oxide formed near the substrate can effectively block the diffusion of the Fe elements from the substrate to the oxide layer. This is mainly due to the fact that the Cr element can react with the O element preferentially over the Fe element, forming a relatively dense protective layer to protect the matrix from the oxidation and erosion of the O element. There are trace fluctuations of the element Fe in the areas where cracks and other defects appear inside the oxide layer. The oxide layer thickness of 444-type ferritic stainless steel is greater than that of the other three types of ferritic stainless steel. No significant changes in the Cr element concentration were found in the matrix near the oxide film, and it was shown that none of the experimental steels experienced excessive volatilization losses of the element Cr.

**Figure 11:**
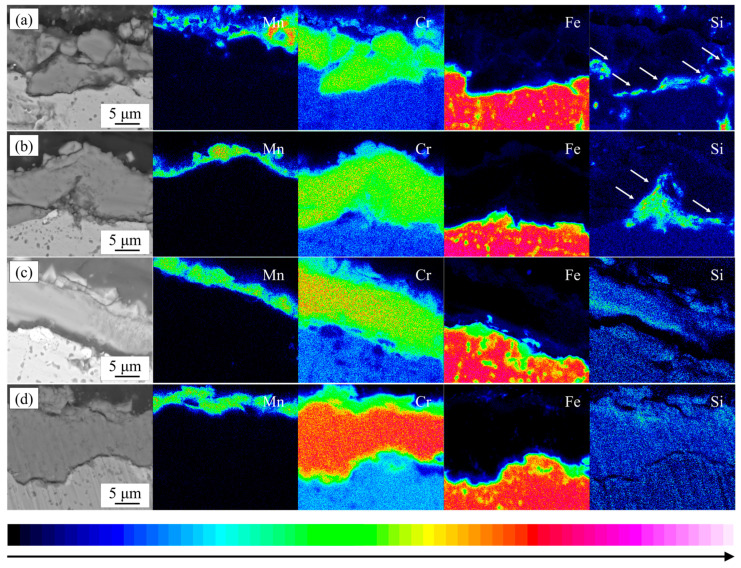
Sectional morphologies and the corresponding EPMA element maps of the oxide layers of the experimental steel after cyclic oxidation in air at 1050 °C for 200 cycles: (**a**) 444; (**b**) 444-Ce; (**c**) 444-0.5W-Ce; (**d**) 444-1W-Ce.

The sectional morphologies and the corresponding EPMA element maps of the oxide layers of the experimental steel after cyclic oxidation in air at 1050 °C for 200 cycles are shown in Figure 11. It can be observed that the element Cr was distributed throughout the inner and outer oxide films and the concentration of Cr in the inner oxide film was higher than its concentration in the outer oxide film. From the distribution of the Mn element, the distribution of the oxidation layer inside and outside the experimental steel can also be clearly seen. The outer oxide layer of 444 ferritic stainless steel is not as continuous and complete as the other three types of stainless steel due to severe spalling. During the oxidation process, drastic temperature changes result in high stress and the formation of defects such as cracks and voids at the interface between the oxide film and the substrate. The diffusion coefficient of Si^4+^ ions in the oxide layer is small, so they tend to occur more at the interface between the matrix and the oxide layer. Enrichment of Si elements could be observed at locations such as cracks and cavities in the oxide film, indicating the formation of Si oxides at these locations. The formation of Si oxide will prevent the repair of defects at the interface between the oxide layer and the substrate, which is not conducive to the long-term service of ferritic stainless steel at high temperatures.

**Figure 12:**
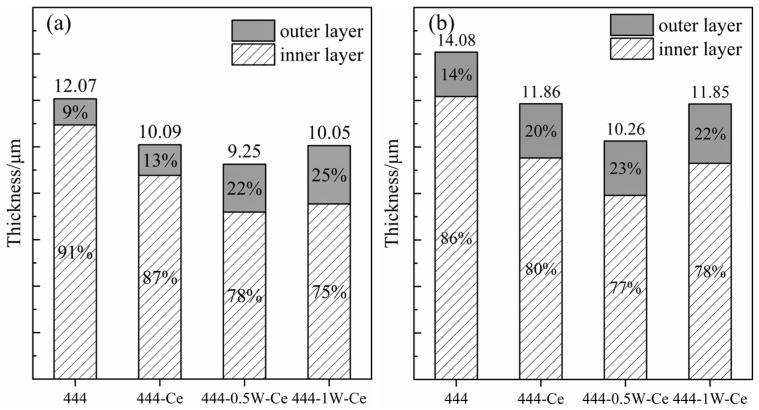
Oxide film composition of experimental steel after cyclic oxidation in air at 1050 °C for 50 and 200 cycles: (**a**) 50 cycles; (**b**) 200 cycles.

Due to the clear reflection of the composition of the oxide layer by the distribution of the Mn and Cr elements, the average thickness of the experimental steel oxide layer and the proportion of the inner and outer oxide layers can be roughly calculated through the element distribution area. Four areas on each specimen were selected for area scanning. The range of distribution of the Cr and Mn elements was measured by using ImageJ software (1.54g.1.8.0.345) and the average thickness of the oxide layer was calculated. The results show that the thickness of the oxide film of 444 ferritic stainless steel was larger than that of the other three experimental steels (shown in Figure 12), and its thickness reached 14.08 μm after cyclic oxidation for 200 cycles. The oxide film of 444-0.5W-Ce steel was the thinnest, with an average thickness of only 10.26 μm. When the W element is added, the proportion of the outer oxide layer will increase. The outer Cr-Mn spinel type oxide layer can reduce the reactive volatilization of Cr oxide, ensuring that the oxide layer maintains a stable element concentration during long-term service and avoiding the occurrence of loose oxide layer due to a poor Cr content.

### 3.5. Strengthening Mechanism of W- and Ce-Containing Ferritic Stainless Steel

**Figure 13:**
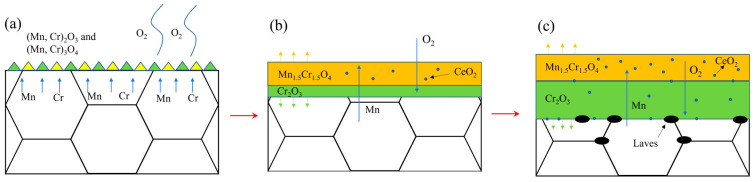
The formation process of inner and outer oxide film of the 444-type ferritic stainless steel in the high-temperature oxidation process. (**a**) Reaction of the matrix surface and oxygen; (**b**) Diffusion of ions in the double oxide film; (**c**) Internal oxidation of ferritic stainless steel and pinning effect of its precipitates on the inner oxide film.

**Figure 14:**
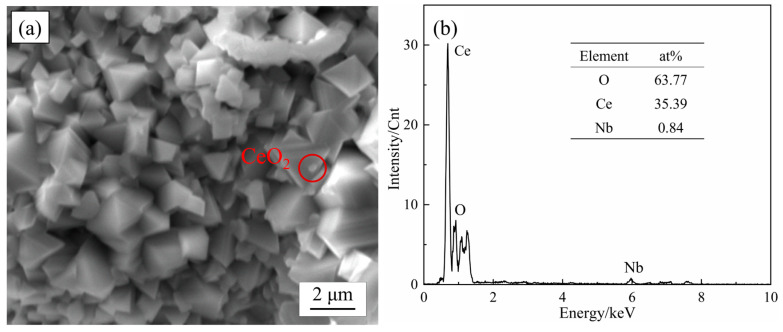
SEM images of rare earth oxides between oxide particles in the oxide film of 444-0.5W-Ce ferritic stainless steel after cyclic oxidation in air at 1050 °C for 200 cycles (**a**) and its EDS analysis (**b**).

The growth of the oxide film is controlled by the diffusion and reaction of reactive elements in the oxide film [32]. In 18Cr ferritic stainless steel, Mn ions exhibit higher diffusivity and a stronger oxygen ion affinity compared to Cr ions in the oxide film. Consequently, during the initial stage of oxidation, the Mn oxides will rapidly occupy the outer positions to form a continuous oxide film (as shown in Figure 13a). Rare earth elements are very active and will form fine rare earth oxide particles in the oxide film. These CeO_2_ oxides serve as nucleation sites for Mn oxides and promote the formation of Mn oxides [33], which can inhibit the further growth of the oxide film. On the other hand, due to the large size of the rare earth elements, the lattice distortion caused by the solid solution of the element Ce in the matrix can increase the degree of lattice openness, which is conducive to the diffusion of reactive elements such as Cr [34] and promotes the formation of the oxide film within Cr_2_O_3_. The formation of the inner oxide film decreases the diffusion rates of Mn and O ions within the film [35], resulting in a reduction in Mn content in the outer oxide layer. This change prompts the transformation of the oxide structure from nodular-like to spinel oxides. The oxide film formed by the Cr-Mn spinel oxides is denser and smoother, which further reduces the growth of the oxide film. As the number of cycles of oxidation continues to increase, the growth of the oxide film gradually stabilizes. At this time, the CeO_2_ oxide formed inside the oxide film (as shown in Figure 14a) can form an oxide plug and have a pinning effect on the oxide film, improving the binding between oxide particles. During the cyclic oxidation, the formation of the oxide layer induces the precipitation of Laves phases at the matrix interface [36]. These Laves phases can inhibit the diffusion of elements at the interface and hinder the inward growth of the oxide film (as shown in Figure 8b–d and Figure 9d), forming a serrated boundary of the inner oxide film. It should be noted that when the addition of the element W is high, the Laves phase at the interface between the matrix and the oxide film will grow and coarsen. Once these Laves phases form large and continuously distributed precipitates bands, it will lead to an increase in the tendency of the oxide film to crack. In severe cases, it led to spallation of the oxide film.

## 4. Conclusions

This study aims to improve the high-temperature cyclic oxidation resistance of 444-type ferritic stainless steel by adding the W and Ce elements. A study was conducted on the high-temperature cyclic oxidation behavior and strengthening mechanism of 444-type modified stainless steel with different W and Ce contents at 1050 °C for different cycles. The following conclusions can be drawn.

(1) The addition of Ce and W significantly reduces the high-temperature cyclic oxidation rate of 444-type ferritic stainless steel. Ce oxides enhances the nucleation of Cr-Mn oxides, refining oxide particle size and stabilizing the oxide film against cracking and spalling.
(2) The element Ce can promote the formation of the inner oxide film. The dense Cr_2_O_3_ oxide film can inhibit the diffusion of Mn^3+^ ions inside the oxide film and reduce the concentration of the Mn element in the oxide of the outer oxide layer. This change can promote the transformation of the outer oxide film from nodular-like oxides to spinel oxides with a better oxidation resistance. The Cr-Mn spinel oxide film can inhibit the diffusion of O^2−^ ions and hinder the inward growth of the inner oxide film.
(3) By promoting Laves phase precipitation at the oxide–matrix interface, which slows interfacial element diffusion and inhibits oxide film growth, W has a great influence on the oxidation behavior of ferritic stainless steel. However, an excessive content of W may result in the coarsening of the Laves phase over time, which could affect the adhesion between the film and the matrix if the phase grows excessively large.

## Figures and Tables

**Table 1:** The chemical composition of the experimental ferritic stainless steels (wt%).

Steel	C	Mn	N	Cr	Mo	Nb	Ti	Si	W	Ce	Fe
444	0.006	0.33	0.008	18.43	1.82	0.436	0.174	0.54	–	–	78.256
444-Ce	0.009	0.32	0.007	19.98	2.05	0.56	0.155	0.52	–	0.048	76.351
444-0.5W-Ce	0.007	0.32	0.008	19.78	1.95	0.44	0.137	0.50	0.58	0.056	76.222
444-1W-Ce	0.01	0.35	0.007	19.87	2.03	0.55	0.141	0.53	1.12	0.053	75.339

**Table 2:** Oxidation rate constants of experimental steel and several similar steels during oxidation in air at different temperatures.

Temperature (°C)	Steel	*K_p_* (mg^2^∙cm^−4^∙h^−1^)
950 °C	444	5.19 × 10^−3^
	444-Ce	9.38 × 10^−4^
	444-0.5W-Ce	9.05 × 10^−4^
	444-1W-Ce	8.71 × 10^−4^
1050 °C	444	1.87 × 10^−2^
	444-Ce	3.07 × 10^−3^
	444-0.5W-Ce	2.76 × 10^−3^
	444-1W-Ce	2.81 × 10^−3^
1100 °C	444	7.53 × 10^−2^
	444-Ce	1.63 × 10^−2^
	444-0.5W-Ce	1.84 × 10^−2^
	444-1W-Ce	1.22 × 10^−2^
900 °C	Crofer 22 H [22]	6.80 × 10^−5^
1000 °C (isothermal oxidation)	Crofer 22 APU [23]	3.71 × 10^−4^
900 °C (isothermal oxidation)	18Cr-Al-Si [24]	3.41 × 10^−4^
950 °C	441 [25]	2.16 × 10^−3^

**Table 3:** Oxidation activation energy of experimental steel in different temperature ranges.

Temperature (°C)	Steel	*Q* (kJ∙mol^−1^)
950–1050 °C	444	246
	444-Ce	279
	444-0.5W-Ce	292
	444-1W-Ce	281

**Table 4:** The EDS results of chemical composition in some areas in Figure 7 and Figure 8.

Area	Chemical Composition (at%)							
	Fe	Cr	Mn	O	Si	Mo	W	Nb
1	−	28.86	−	61.83	9.31	−	−	
2	−	26.17	16.59	25.63	−	−	−	
3	0.93	38.34	−	57.24	−	−	−	
4	56.76	13.95	−	−	2.54	18.86	−	7.89
5	58.27	8.95	−	−	3.07	8.98	8.45	12.28
6	45.19	8.92	−	−	3.51	5.26	18.08	19.04

## Data Availability

Data are contained within the article.
